# PIWI-Interacting RNA Pathway Genes: Potential Biomarkers for Clear Cell Renal Cell Carcinoma

**DOI:** 10.1155/2022/3480377

**Published:** 2022-03-01

**Authors:** Baoli Heng, Xuexia Xie, Wenjuan Zeng, Haomin Li, Liping Shi, Wencai Ye, Fanyu Wu

**Affiliations:** ^1^Yingde Center, Institute of Kidney Surgery, Jinan University, Guangdong, China; ^2^Postdoctoral Mobile Station, The First Clinical Medical College of Jinan University, Guangzhou, China; ^3^Department of Urology, People's Hospital of Yingde City, Yingde, China; ^4^Department of Urology, The First Affiliated Hospital of Jinan University, No. 613, Huangpu Road, Guangzhou, China; ^5^Department of Obstetrics and Gynecology, The First Affiliated Hospital of Jinan University, No. 613, Huangpu Road, Guangzhou, China; ^6^College of Pharmacy, Jinan University, Guangzhou 510632, China; ^7^Guangdong Province Key Laboratory of Pharmacodynamic Constituents of Traditional Chinese Medicine and New Drugs Research, Jinan University, Guangzhou 510632, China

## Abstract

**Background:**

Clear cell renal cell carcinoma (ccRCC) is one of the most lethal malignancies in the urinary system, yet effective diagnostic and prognostic markers are lacking. Recently, several of piRNA pathway genes have been reported to be associated with cancer diagnosis and prognosis, but their role in ccRCC is still unclear.

**Methods:**

We analysed the expression of 27 piRNA pathway genes in 539 kidney renal clear cell carcinoma (KIRC) and 72 nontumor tissue samples (data from TCGA), and 12 mRNAs were significantly different. The aim was to sift the piRNA pathway genes that are correlated with ccRCC patient survival and to construct a piRNA pathway gene risk prognostic model using Kaplan-Meier survival curve and ROC curve, respectively.

**Results:**

5 piRNA pathway genes (TDRD7, GPAT2, PLD6, SUV39H1, and DOM3Z) were picked out and used to construct the piRNA pathway gene risk model. Kaplan-Meier survival curve analysis showed that compared with that of the low-risk group of ccRCC patients, the OS of the high-risk group of ccRCC patients was significantly reduced. The predictive performance of the prognostic risk model was measured using a ROC curve, which individually showed AUC values for 1 year of 0.707, for 3 years of 0.713, and for 5 years of 0.701. Moreover, the mRNA and protein expression levels of TDRD7 were overexpressed in the ccRCC datasets (data from our cohort, TCGA, GEO, and CPTAC) and ccRCC cell lines, and the expression levels correlated with the clinicopathological characteristics in ccRCC. The Tumor Immune Estimation Resource (TIMER) showed that the mRNA expression level of TDRD7 was positively related to tumor immune infiltrating cells (TICs) in ccRCC. Mechanistically, gene set enrichment analysis (GSEA) was performed to uncover the mechanism of TDRD7 in ccRCC. In summary, the piRNA pathway genes,especially TDRD7, may be potential cancer diagnostic and prognostic biomarkers of ccRCC.

## 1. Introduction

Renal cell carcinoma (RCC) is one of the most common tumors of the urinary system. In 2020, approximately 73,750 new cases and 14,8308 deaths occurred in the USA [1]. RCC is composed of various histological and molecular subtypes, of which clear cell renal cell carcinoma (ccRCC) is the most common, accounting for approximately 70-80% [2]. At present, for the diagnosis of RCC, the most frequently used technique is computed tomography (CT). Due to its resistance to radiotherapy and conventional chemotherapy, surgery is the most effective treatment for localized RCC [3]. Unfortunately, approximately 30% of patients have distant metastases at the time of diagnosis [4]. These patients lose their best treatment and have a worse prognosis. Therefore, there is a need for an urgent solution to find new diagnostic and therapeutic targets to reduce the mortality rate of ccRCC.

PIWI-interacting RNAs (piRNAs) are small noncoding RNAs (ncRNAs) of 24-32 nucleotides in length that specifically interact with PIWI proteins in the Argonaute protein family [5]. piRNAs exist in germ cells and somatic cells and have crucial functions for instance inhibition of transposable elements (TEs) and epigenetic regulation of gene expression. piRNA formation involves three pathways: primary processing, secondary processing (ping-pong cycle), and TE transcriptional silencing [6]. The primary processing of piRNA can occur in germline cells and their surrounding somatic cells, but the ping-pong cycle can only occur in germline cells [7, 8]. piRNAs are processed in a conserved perinuclear structure called the nuage, which contains piRNA pathway proteins, including the Piwi branch of the Argonaute family of proteins, as well as some Tudor domain proteins, RNA helicases, and nucleases. Tudor domain containing 7 (TDRD7) is a Tudor domain protein that is mainly expressed in germline cells and colocalizes with other piRNA pathway components [9]. Its function in the piRNA pathway is to regulate inverted transposons in Drosophila germlines [10].

With the development of next-generation sequencing, piRNAs are found to play important roles in RCC. For example, Iliev et al. found that the higher the level of piR-823 in tumor tissue, the shorter the disease-free survival, and the higher the level of piR-823 in serum, the higher the clinical stage of RCC. piR-823 shows potential for early diagnosis of tumors [11]. Zhao et al. measured the expression of two kinds of mitochondrial piRNAs (piR-34536 and piR-51810) in the tissues and serum of ccRCC patients [12]. The results showed that the expression levels of piR-34536 and piR-51810 in ccRCC tissues were significantly lower than those in normal tissues, and the level of mitochondrial piRNA was negatively correlated with the prognosis of ccRCC patients. However, the function of piRNA pathway genes in ccRCC remains unclear.

In this study, we explored the diagnostic and prognostic value of piRNA pathway genes in ccRCC tumors via constructing a piRNA pathway gene risk prognostic model (using data derived from TCGA). We found that the piRNA pathway gene risk model could be an independent prognostic factor in ccRCC. Then, we found that the piRNA pathway gene TDRD7 was overexpressed in ccRCC. High TDRD7 expression was relevant to tumor progression and immune infiltrating cells in patients with ccRCC. Our data revealed that TDRD7 may provide a reliable biomarker for the diagnosis and prognosis of ccRCC.

## 2. Materials and Methods

### 2.1. The Cancer Genome Atlas (TCGA)

TCGA (https://genomecancer.ucsc.edu/), which is a large, free tumor data portal of the human genome project, contains the RNA sequence and clinical and pathological information of ccRCC patients. Data on patients with ccRCC in TCGA included 539 ccRCC tumor specimens and 72 normal renal specimens. Through R software (https://www.r-project.org/), the expression of RNA sequences was downloaded and matched with clinicopathological information. All raw data were normalized, log2 transformed, and data with an average count < 1 gene were eliminated. A ∣log2 − fold change | >0.5 and a false discovery rate (FDR) < 0.05 as the cut-off values were considered statistically significant.

### 2.2. The Gene Expression Omnibus (GEO) Database and Our Cohort Data

The GEO database, a synthetic gene expression library maintained by the National Center of Biotechnology Information (NCBI) (https://www.ncbi.nlm.nih.gov/geo/), is one of the world's largest collections of gene chips. The TDRD7 raw mRNA expression matrix in four ccRCC cohorts (GSE53757, GSE66270, GSE68417, and GSE76351 datasets) was obtained by the “Limma” package. The transcriptome-sequencing data of 34 pairs ccRCC and adjacent normal tissues were referred to in our previous study [13].

### 2.3. The Human Protein Atlas and Clinical Proteomic Tumor Analysis Consortium (CPTAC) Database

The Human Protein Atlas provides proteome and transcriptome information on a wide range of different human samples. Immunohistochemistry of TDRD7 protein in normal and ccRCC tissues was derived from this website https://www.proteinatlas.org/search/TDRD7 . The clinical data and matched TDRD7 protein expression in the CPTAC ccRCC database were performed via the UALCAN website (http://ualcan.path.uab.edu/).

### 2.4. Prognostic Model Construction and Validation

We randomly divided 530 ccRCC patients into two groups: the training group (*n* = 266) and the test group (*n* = 264) (data from TCGA). We calculated the risk score for each patient using the regression coefficients of individual piRNA pathway genes and the expression values of each selected piRNA pathway gene obtained from the multivariate Cox risk model.

(1) $$\begin{array}{c} \text{The risk score}=\sum \text{r}\text{egression coefficient }\left(\text{genei}\right)\times \text{expression value of }\left(\text{genei}\right)\\ i=1,2,\cdots ,n \end{array}$$

Risk scores were calculated from a linear combination of the relative gene expression levels multiplied by the regression coefficients. The regression coefficients were obtained by multiple Cox analyses and represented the relative weights of genes.

### 2.5. TIMER Database Analysis

TIMER (https://cistrome.shinyapps.io/timer/) is an online dataset used to assess clinical associations, mutations, and the relationships between somatic copy number alterations (SCNAs) and the invasion of seven immune cells in different cancer types. The Spearman correlation test was used to analyse the correlation between TDRD7 expression and the level of immune cell infiltration.

### 2.6. Survival Analysis and Receiver Operator Characteristic (ROC) Curve Analysis of piRNA Pathway Genes

From the online database Kaplan-Meier Plotter (http://kmplot.com/analysis/), overall survival (OS) data were downloaded, where the cut-off value was the median. To verify the diagnostic value, the area under the ROC curve (AUC) was calculated by GraphPad Prism 8 (survival data from the TCGA database) to evaluate the diagnostic value. All *P* values < 0.05 were considered statistically significant.

### 2.7. Gene Set Enrichment Analysis (GSEA)

According to the median expression level of TDRD7, the samples in the ccRCC patient datasets were separated into high and low expression groups, and GSEA (http://www.gsea-msigdb.org/gsea/index.jsp) was used to examine whether genes that were enriched in both groups were involved in life processes in a meaningful way. FDR (value) < 0.25 and *P* < 0.05 were considered statistically significant.

### 2.8. Cell Culture

ccRCC cell lines (786O, ACHN, and A498) and normal kidney tubular epithelial cell lines (HK-2) were obtained from the American Type Culture Collection (ATCC) and cultured in medium with 10% fetal bovine serum (FBS) at 37°C and 5% CO2.

### 2.9. RNA Extraction and Quantitative Real-Time PCR

Extraction of total RNA with TRIzol Reagent (Invitrogen; Thermo Fisher Scientific). cDNA was reverse transcribed using the PrimeScript™ RT reagent Kit (Takara, Japan). qPCR was performed using SYBR Green Real-time PCR Master Mix (Vazyme, Nanjing, China). The primer sequences of TDRD7 and *β*-actin were as follows (5′-3′): TDRD7, forward TCTGAGAAGTGTGCCAGCAG and reverse TTTGACGAGCCACAAGCTGA; and *β*-actin, forward TTTGAGACCTTCAACACCCCA and reverse TTTCGTGGATGCCACAGGA.

### 2.10. Statistical Analysis

All data were analysed using SPSS software (Version 26.0; IBM, Armonk, NY, USA). First, the normality and homogeneity of variance were tested. When normality and homogeneity of variance were met, comparisons between multiple groups of data were performed by ANOVA, and two-group comparisons were performed using Student's *t*-test. The results are expressed as the mean ± standard deviation (mean s ± SD), and *P* < 0.05 indicates that the difference is statistically significant.

## 3. Results

### 3.1. Identification of Differentially Expressed piRNA Pathway Genes in ccRCC

**Figure 1:**
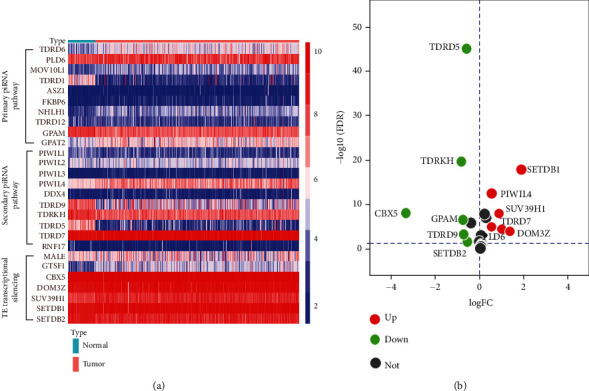
Differential expression of piRNA pathway genes in ccRCC tissue samples. (a) Heat map of the differential expression of 27 piRNA pathway genes in ccRCC tissue samples (*n* = 539) compared with normal kidney samples (*n* = 72). (b) Volcano plot of 27 piRNA pathway genes in ccRCC tissue samples. The red dot represents upregulated genes, the green dot represents downregulated genes, and the grey dot represents unchanged genes. FC: fold change; FDR: false discovery rate.

A total of 539 ccRCC and 72 normal kidney tissue specimens from TCGA were contained in this study. We used R software to filter 27 piRNA pathway genes and identified 12 piRNA pathway genes that were differentially expressed. This included 6 upregulated (SETDB1, DOM3Z, PIWIL4, SUV39H1, PLD6, and TDRD7) and 6 downregulated (CBX5, TDRKH, TDRD5, GPAM, TDRD9, and SETDB2) piRNA pathway genes (FDR < 0.05, ∣log_2_FC | >0.5) (Figure 1). ∣log_2_FC | >0.5 was set as the lower limit of expression abundance of piRNA pathway genes in ccRCC.

### 3.2. Construction of a Prognostic Model of piRNA Pathway Genes

**Figure 2:**
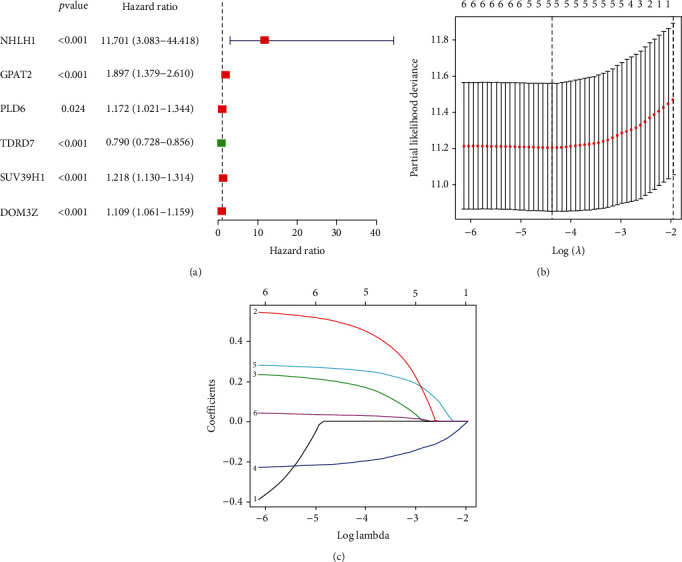
Identify survival-related piRNA pathway genes in ccRCC patients and development of a prognostic model. (a) The risk ratio forest plot showed the prognostic value of 6 candidate genes screened out by univariate Cox regression. (b) and (c) LASSO coefficient profiles of 5 piRNA pathway genes; The partial likelihood deviance plot displayed the minimum number corresponds to the covariates utilized for multivariate Cox analysis. 1, NHLH1; 2, GPAT2; 3, PLD6; 4, TDRD7; 5, SUV39H1; 6, DOM3Z.

To build a prognostic risk model, univariate regression analysis was performed to identify six piRNA pathway genes (Figure 2(a)). The LASSO Cox regression model was used to escape excessive model fitting through multivariate Cox regression analysis (Figures 2(b) and 2(c)). The risk scores are shown below:

(2) $$\begin{array}{c} \text{Risk score}=\left(0.4862\times \text{Exp GPAT}2\right)+\left(0.1898\times \text{Exp PLD}6\right)+\left(-0.2101\times \text{Exp TDRD}7\right)+\left(0.2628\times \text{Exp SUV}39\text{H}1\right)+\left(0.0315\times \text{Exp DOM}3\text{Z}\right). \end{array}$$

### 3.3. Evaluation of the piRNA Pathway Gene Prognostic Risk Model

**Figure 3:**
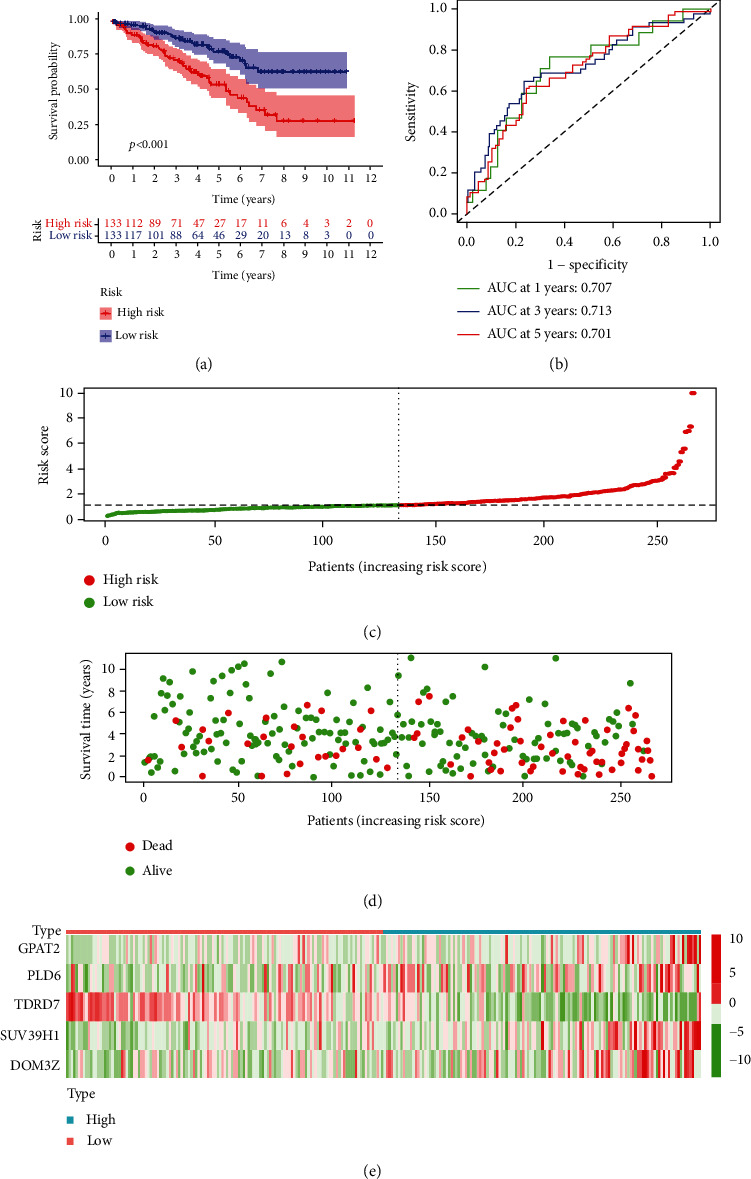
Risk score analysis of the 5 piRNA pathway genes prognostic risk model in the training group ccRCC patients. (a) Kaplan-Meier survival curve analysis shows the overall survival of high- (*n* = 133) and low-risk (*n* = 133) training group ccRCC patients based on the median risk score calculated using the 5 piRNA pathway genes prognostic risk model. (b) Time-dependent ROC curve analysis shows the prognostic performance of the 5 piRNA pathway genes prognostic risk model in predicting 1-year, 3-year, and 5-year survival times of the high- and low-risk training group ccRCC patients. (c) Risk curve analysis of the 5 piRNA pathway genes prognostic risk model in high- and low-risk training group ccRCC patients. (d) Scatter plots show the survival status of the 5 piRNA pathway genes prognostic risk model in the training group ccRCC patients. (e) The heat map shows the expression of the 5 piRNA pathway genes prognostic risk model in high- and low-risk training group ccRCC patients.

**Figure 4:**
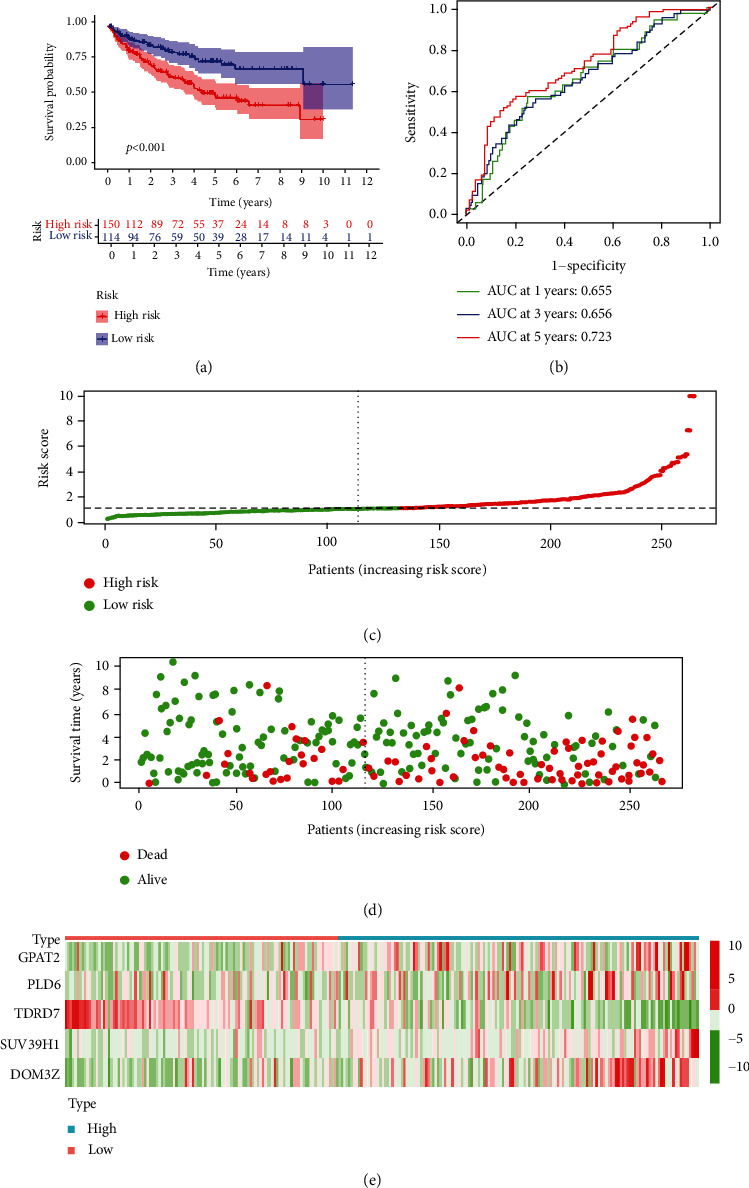
Risk score analysis of the 5 piRNA pathway genes prognostic risk model in the testing group ccRCC patients. (a) Kaplan-Meier survival curve analysis shows the overall survival of high- (*n* = 150) and low-risk (*n* = 114) testing group ccRCC patients based on the median risk score calculated using the 5 piRNA pathway genes prognostic risk model. (b) Time-dependent ROC curve analysis shows the prognostic performance of the 5 piRNA pathway genes prognostic risk model in predicting 1-year, 3-year, and 5-year survival times of the high- and low-risk testing group ccRCC patients. (c) Risk curve analysis of the 5 piRNA pathway genes prognostic risk model in high- and low-risk testing group ccRCC patients. (d) Scatter plots show the survival status of the 5 piRNA pathway gene prognostic risk model in the testing group ccRCC patients. (e) The heat map shows the expression of the 5 piRNA pathway gene prognostic risk model in high- and low-risk testing group ccRCC patients.

We first analysed 266 patients separated into high- and low-risk groups (based on the median risk score) in the training group. Kaplan-Meier survival curve analysis showed that compared with that of the low-risk group of ccRCC patients, the OS of the high-risk group of ccRCC patients was significantly reduced (Figure 3(a)). The predictive performance of the prognostic risk model was measured using a ROC curve, which individually showed AUC values for 1 year of 0.707, for 3 years of 0.713, and for 5 years of 0.701 (Figure 3(b)). We then analysed their distribution by ranking the risk scores of patients for OS (Figure 3(c)). The dot plots show the OS status of a single patient with ccRCC (Figure 3(d)). The heat maps show the expression levels of the risk genes in the training group (Figure 3(e)). These results demonstrate the moderate performance of the prognostic prediction model based on piRNA pathway gene characteristics. Then, we used the same methods in the test group and evaluated the predictive performance of the prognostic risk model. This result is similar to the above result (Figure 4). These results prove the stable performance of the prognostic prediction model based on piRNA pathway genes.

### 3.4. The Prognostic Model Based on the piRNA Pathway Was an Independent Risk Factor for OS in ccRCC Patients

**Figure 5:**
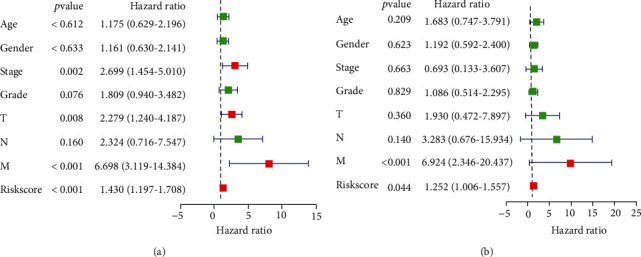
Independent prognostic factor evaluation. (a) Univariate cox regression analysis of the training dataset (TCGA). (b) Multivariate cox regression analysis of the training dataset (TCGA).

The TCGA ccRCC dataset was used to verify the features we identified, and miscellaneous clinical parameters were used as independent prognostic factors for ccRCC by univariate and multivariate Cox regression analysis. Univariate analysis showed that the piRNA pathway gene risk score, stage, T classification, and metastasis classification were markedly correlated with OS (all *P* < 0.05). Moreover, multivariate analysis indicated that the piRNA pathway gene risk score and metastasis classification were significantly related to OS (all *P* < 0.05). Hence, the piRNA pathway genes could be an independent prognostic factor for patients with ccRCC (Figure 5, Table S1).

### 3.5. Survival and ROC Curve Analysis of the Five piRNA Pathway Genes

**Figure 6:**
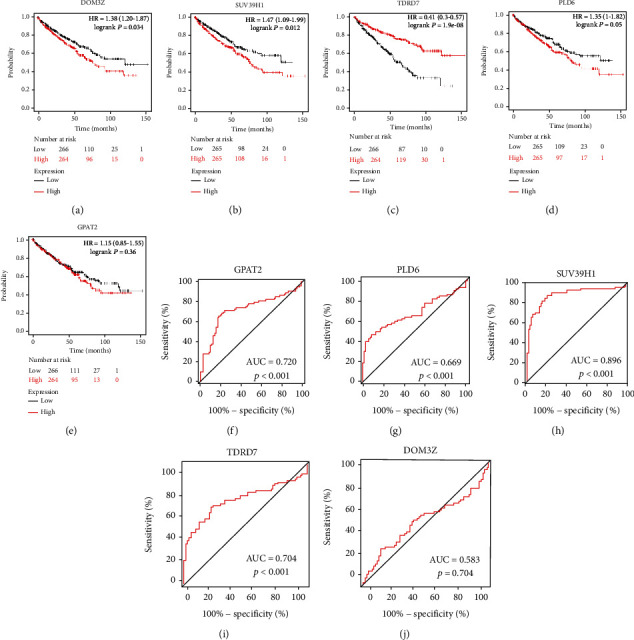
Overall survival (OS) Kaplan-Meier and receiver operator characteristic (ROC) curves of 5 piRNA pathway genes in ccRCC. The survival analyses of (a) DOM3Z (*P* = 0.034), (b) SUV39H1 (*P* = 0.012), (c) TDRD7 (*P* < 0.001), (d) PLD6 (*P* = 0.05), and (e) GPAT2 (*P* = 0.360); the ROC curve plots for (f) GPAT2 (AUC = 0.720, *P* < 0.001), (g) PLD6 (AUC = 0.669, *P* < 0.001), (h) SUV39H1 (AUC = 0.896, *P* < 0.001), (i) TDRD7 (AUC = 0.704, *P* < 0.001), and (j) DOM3Z (AUC = 0.583, *P* = 0.704) genes in ccRCC are shown.

We enforced OS survival analysis to estimate the prognostic influence of the expression of the five piRNA pathway genes in ccRCC patients by the Kaplan-Meier Plotter website. The results showed that high expression of DOM3Z (*P* = 0.034) and SUV39H1 (*P* = 0.012) predicted poor OS and that low expression of TDRD7 (*P* < 0.001) predicted poor OS (Figures 6(a)–6(c)). However, the expression of PLD6 (*P* = 0.05) and GPAT2 (*P* = 0.36) was not significantly related to OS (Figures 6(d) and 6(e)). We then used ROC curves to estimate the diagnostic role of the five piRNA pathway genes in ccRCC. A total of 72 pairs of ccRCC tissues and normal renal tissues from TCGA were used as a control to produce this ROC curve. The results showed that GPAT2 (AUC = 0.720, *P* < 0.001) (Figure 6(f)), PLD6 (AUC = 0.669, *P* < 0.001) (Figure 6(g)), SUV39H1 (AUC = 0.896, *P* < 0.001) (Figure 6(h)), and TDRD7 (AUC = 0.704, *P* < 0.001) (Figure 6(i)) could effectively differentiate ccRCC patients. However, the expression of DOM3Z was not significantly associated with OS (Figure 6(j)). Overall, the results indicated that the expression of the five piRNA pathway genes was markedly associated with the prognosis and diagnosis of ccRCC patients and could be useful as a biomarker for the prognosis of ccRCC patients and as diagnostic targets for ccRCC. According to previous results, we believe that TDRD7 is a key gene of the piRNA pathway in ccRCC. Thus, we chose TDRD7 for further study.

### 3.6. Expression Levels of TDRD7 in ccRCC Patients

**Figure 7:**
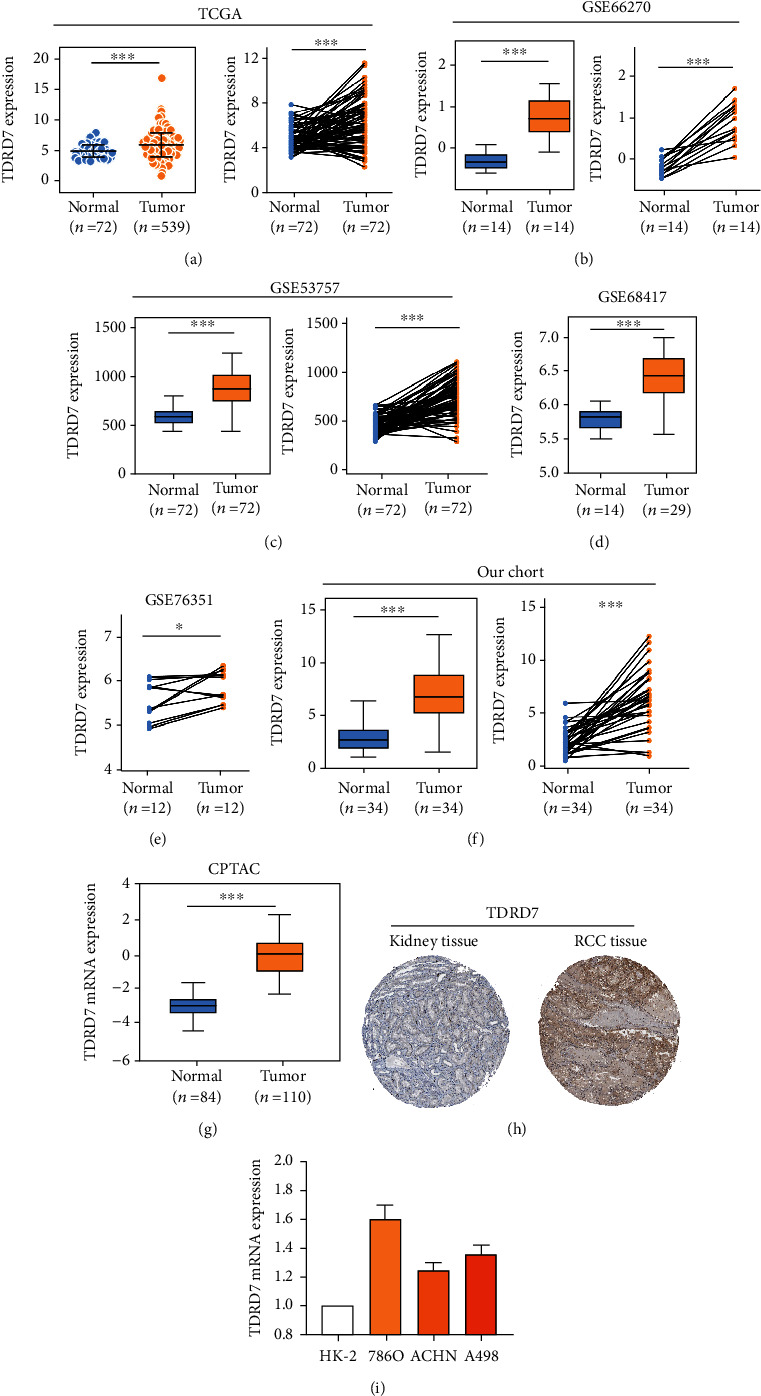
TDRD7 was overexpressed in ccRCC. (a) The left panel shows the TDRD7 mRNA expression was compared between normal tissues (*n* = 72) and ccRCC (*n* = 539) tissues in TCGA database; the right panel shows the TDRD7 expression was compared between normal tissues (*n* = 72) and matched ccRCC (*n* = 72) tissues in TCGA database. (b)–(e) The expression of TDRD7 mRNA in ccRCC and normal tissues in GEO database, including GSE66270 (*n* = 28), GSE53537 (*n* = 144), GSE68417 (*n* = 43), and GSE76351 (*n* = 24). (f) The expression of TDRD7 in our ccRCC chort patient's transcriptome-sequencing data. (g) The expression of TDRD7 protein in ccRCC tissues and normal kidney tissues (data from CPTAC). (h) The level of TDRD7 protein in RCC tissue was higher than that in normal kidney tissue in the Human Protein Atlas (Antibody CAB020800, 10X). (i) The expression of TDRD7 mRNA in ccRCC cell lines (786O, ACHN, and A498) and human normal kidney epithelial cell (HK-2). ^∗^*P* < 0.05; ^∗∗^*P* < 0.01; ^∗∗∗^*P* < 0.001.

Transcriptome sequencing data from the TCGA dataset were used to assess the expression level of TDRD7 in ccRCC. TCGA results revealed that the TDRD7 expression level was upregulated in ccRCC tissues (Figure 7(a)). We used microarray data from GEO (GSE66270, GSE53747, GSE66270, and GSE68417) to further confirm the expression of TDRD7 in ccRCC. The results showed that TDRD7 was also highly expressed in ccRCC tissues (Figures 7(b)–7(e)). Moreover, similar results were observed in our ccRCC patient cohort transcriptome-sequencing data (Figure 7(f)). In addition, compared with that of normal tissues, the protein expression level of TDRD7 in ccRCC tissues was significantly higher (data from the CPTAC) (Figure 7(g)). The TDRD7 immunohistochemistry (IHC) results were obtained from the Human Protein Atlas and were in line with the results obtained from the CPTAC (Figure 7(h)). Moreover, qRT-PCR detected that all of the ccRCC cell lines exhibited high TDRD7 expression levels in contrast to that of the normal kidney epithelial cell line (HK-2) (Figure 7(i)).

### 3.7. Clinical Features Related to TDRD7 Expression in ccRCC

**Figure 8:**
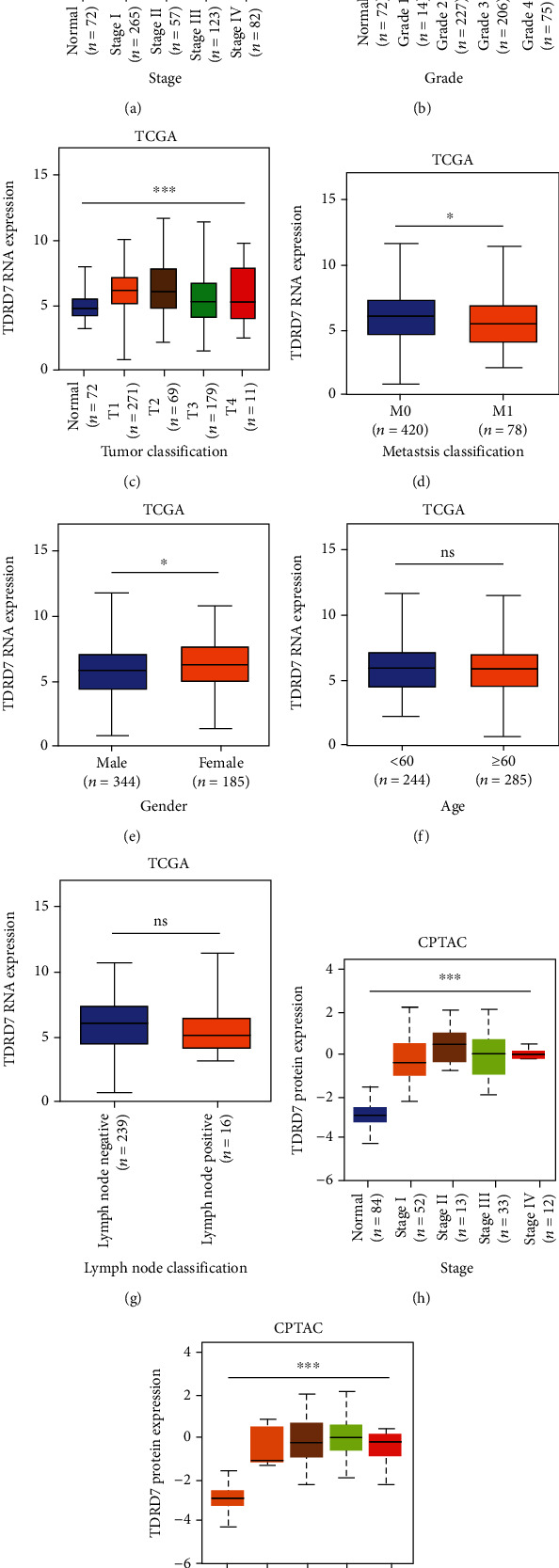
TDRD7 expression was associated with clinicopathological characteristics of patients with clear cell renal cell carcinoma (ccRCC). The TDRD7 mRNA expression level for the patient characteristics of (a) stage, (b) grade, (c) T classification, (d) metastasis classification, (e) gender, (f) age, and (g) lymph node classification. The TDRD7 protein expression level for the patient characteristics of (h) pathologic stage and (i) grade. The data are presented as the means ± SD. ^∗^*P* < 0.05; ^∗∗∗^*P* < 0.001; NS: no significance.

To further identify the underlying role of TDRD7 based on clinical data, we reviewed the clinical data of the TCGA ccRCC patients. Clinical pathological parameters included the individual cancer stages, TNM classification, grade, age, and sex of patients. The expression of TDRD7 was markedly upregulated in ccRCC classified as stages I–IV (Figure 8(a)), grades 1–4 (Figure 8(b)), or T classification 1-4 compared with that of normal renal tissues (Figure 8(c)). Moreover, low expression of TDRD7 mRNA was markedly correlated with metastasis (Figure 8(d)) and sex (Figure 8(e)). Neither the correlation between TDRD7 expression and age (Figure 8(f)) nor that between TDRD7 expression and N classification (Figure 8(g)) in patients with ccRCC was significant. The N classification result may be caused by the small sample size (only 16 patients were lymph node-positive). We further studied the correlation between the TDRD7 protein expression level of ccRCC patients and the abovementioned clinicopathological characteristics. The results showed that the increase in TDRD7 protein expression was related to tumor stage and tumor grade (Figures 8(h) and 8(i)).

### 3.8. Correlation between TDRD7 Expression and Tumor Immune Infiltrating Cells (TICs)

**Figure 9:**
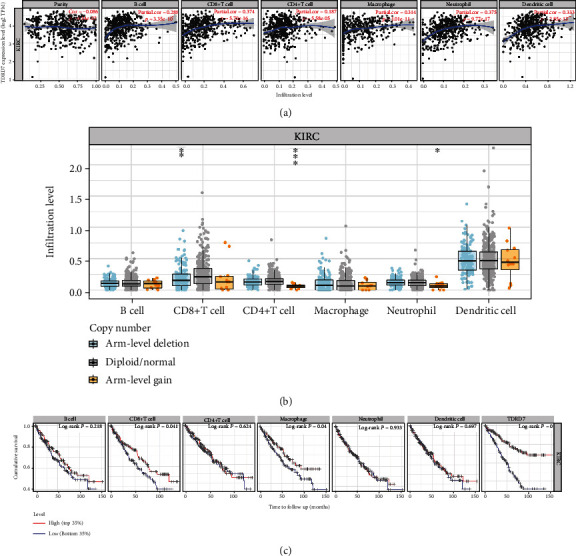
Correlation between TDRD7 and tumor immune infiltrating cells. (a) Correlation between the expression of TDRD7 and immune infiltrating cells in clear cell renal carcinoma (ccRCC). (b) TDRD7 CNV affects the infiltrating levels of CD4_+_ T cell, CD8_+_ T cell, and neutrophils cell in ccRCC. (c) Kaplan-Meier plots of immune infiltration and TDRD7 expression levels in ccRCC. ^∗^*P* < 0.05; ^∗∗^*P* < 0.01; ^∗∗∗^*P* < 0.001. ns: no significant.

We analysed the relationship between TDRD7 expression and TICs in ccRCC using the TIMER database. The results showed that B cells (*r* = 0.288, *P* = 3.35*E* − 10), CD8^+^ T cells (*r* = 0.374, *P* = 5.70*E* − 16), CD4^+^ T cells (*r* = 0.187, *P* = 5.58*E* − 5), macrophages (*r* = 0.314, *P* = 1.01*E* − 11), neutrophils (*r* = 0.375, *P* = 9.77*E* − 17), and dendritic cells (*r* = 0.333, *P* = 2.85*E* − 13) were positively correlated with the expression levels of TDRD7 (Figure 9(a)). Moreover, we found that TDRD7 copy number variation (CNV) was closely related to the degree of infiltration of CD8^+^ T cells, CD4^+^ T cells, and neutrophils (Figure 9(b)). We further generated Kaplan-Meier curves to study the difference in survival between TDRD7 expression and immune infiltrating cells. We found that CD8^+^ T cell infiltration (*P* = 0.041), macrophages (*P* = 0.040), and TDRD7 expression (*P* < 0.001) were markedly associated with ccRCC prognosis (Figure 9(c)).

### 3.9. GSEA for Identification of TDRD7-Related Signalling Pathways in ccRCC

**Figure 10:**
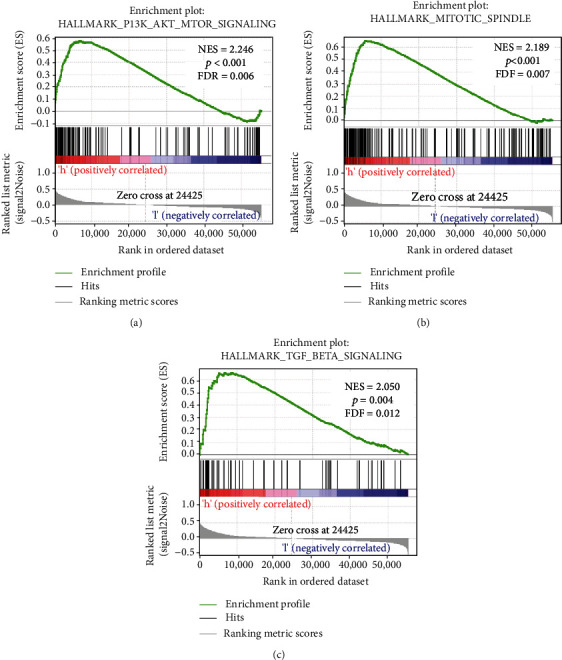
Gene Set Enrichment Analysis (GSEA) of TDRD7 in ccRCC. Pathway enriched in the PI3K/AKT/mTOR signalling pathway (a), mitotic spindle signalling (b), and TGF-*β* signalling (c).

To further validate the relevant signalling pathways activated in ccRCC, we performed GSEA by comparing the high and low TDRD7 expression groups. GSEA showed positive enrichment of a large number of genes in the TDRD7 high expression group, including the PI3K/AKT/mTOR signalling pathway (normalized enrichment score (NES) = 2.25, FDR = 0.006, Figure 10(a)), mitotic spindle signalling pathway (NES = 2.19, FDR = 0.007, Figure 10(b)), and TGF-*β* signalling pathway (NES = 2.05, FDR = 0.012, Figure 10(c)). Together, these results show that TDRD7 might affect tumor proliferation and metastasis and inhibit apoptosis.

## 4. Discussion

Currently, with the improvement in ccRCC diagnostic technology, the diagnostic rate of ccRCC has been greatly advanced. However, a large number of patients still fail to obtain the best treatment due to a lack of early clinical symptoms and sensitive biomarkers. According to reports, a host of piRNAs play an important role in ccRCC [11, 14, 15]. piRNA pathway genes are essential for the formation of piRNAs. However, few studies have focused on the mechanism of piRNA pathway genes and their roles in ccRCC. Therefore, we explored which piRNA pathway genes can be used as novel effective prognostic biomarkers for patients with ccRCC. In this study, the expression of twenty-seven piRNA pathway genes was analysed in ccRCC using the TCGA database, and then, five of the genes were used to build a prognostic risk model. We then selected TDRD7 for further verification.

TDRD7 is a member of the Tudor domain RNA binding (TDRD) protein family. The TDRD protein family, named because it contains one or more Tudor domains, is an evolutionarily conserved family of methylated arginine binding proteins [16, 17]. The TDRD protein family is involved in the formation of the nuage, the piRNA pathway in the gametes, and the occurrence of cancer [18–23]. TDRD7 contains three helix-turn-helix (HTH) domains and two Tudor domains, is widely expressed in the germline and testis, and is associated with reverse transposon inhibition of long interspersed nuclear elements-I (LINE-I) [10]. In our study, by differential gene expression analysis, we found that TDRD7 is highly expressed in ccRCC samples compared with normal kidney specimens. However, when compared the expression of TDRD7 in ccRCC samples with different grades and stages, we noticed that TDRD7 is upregulated in lower malignancy or later stages of ccRCC. Moreover, we found that ccRCC patients with lower TDRD7 expression had a poorer prognosis. These controversial findings obfuscate the role of CHAC1 in the initiation or progression of KIRC [24]. Relevant literature has confirmed that tumor immune infiltration cells are associated with the prognosis of ccRCC patients [25, 26]. The TIMER database showed that the expression level of TDRD7 in ccRCC was positively correlated with the expression levels of tumor immune infiltration cells. This result indicated that TDRD7 may be involved in the immune response of the ccRCC tumor microenvironment. Survival analysis also found that ccRCC patients with low expression of CD8^+^ T cells and macrophages had a worse prognosis. Above, we mentioned that TDRD7 is upregulated in ccRCC tissues, but its high expression is related to a good prognosis in ccRCC. We hypothesized that the overexpression of TDRD7 promotes the infiltration of CD8^+^ T cells and macrophages in ccRCC and ultimately slows tumor progression.

To further confirm the potential mechanism of TDRD7 in ccRCC, GSEA was carried out and showed that the high expression of TDRD7 was related to the PI3K/Akt/mTOR signalling, mitotic spindle signalling, and TGF-*β* signalling pathways. Previous studies have demonstrated that TGF-*β* induced epithelial to mesenchymal transition (EMT) in hepatocellular carcinoma (HCC) through the PI3K/Akt/mTOR signalling pathways [27]. Hence, these results have shown that TDRD7 may regulate the oncogenesis of ccRCC via the TGF-*β*/PI3K/Akt/mTOR signalling pathways. Of course, this study also had several limitations. We only predicted that TDRD7 could be used as a predictor and diagnostic marker through the database, while functional experiments of TDRD7 were lacking, so there was no further verification of our hypothesis in ccRCC.

## 5. Conclusions

In summary, we first found that piRNA pathway genes have different expression levels in ccRCC. Then, we determined that the piRNA pathway gene risk model could be an independent prognostic factor in ccRCC. Furthermore, we chose the piRNA pathway gene TDRD7 for further study. Our results indicated that decreased expression of TDRD7 may be useful in predicting the poor prognosis of patients with ccRCC and may inhibit tumor immune cell infiltration in ccRCC. Moreover, this study revealed that TDRD7 could become a potential diagnostic and prognostic target for ccRCC.

## Data Availability

The data used to support the findings of this study are available from the corresponding author upon request.

## Supplementary Materials

Supplementary Materials Table S1: univariate and multivariate Cox regression analysis of clinical factors and the piRNA pathway gene risk score for overall survival.

## References

[1] Siegel R. L., Miller K. D., Jemal A. (2020). Cancer statistics, 2020. *CA: a Cancer Journal for Clinicians*.

[2] Nabi S., Kessler E. R., Bernard B., Flaig T. W., Lam E. T. (2018). Renal cell carcinoma: a review of biology and pathophysiology. *F1000Res*.

[3] Chen V. J., Hernandez-Meza, Agrawal (2019). Time on therapy for at least three months correlates with overall survival in metastatic renal cell carcinoma. *Cancers*.

[4] Choueiri T. K., Motzer R. J. (2017). Systemic therapy for metastatic renal-cell carcinoma. *The New England Journal of Medicine*.

[5] Xiao Y., Ke A. (2016). PIWI takes a giant step. *Cell*.

[6] Iwasaki Y. W., Siomi M. C., Siomi H. (2015). PIWI-interacting RNA: its biogenesis and functions. *Annual Review of Biochemistry*.

[7] Ku H. Y., Lin H. (2014). PIWI proteins and their interactors in piRNA biogenesis, germline development and gene expression. *National Science Review*.

[8] Cox D. N., Chao A., Lin H. (2000). Piwi encodes a nucleoplasmic factor whose activity modulates the number and division rate of germline stem cells. *Development*.

[9] Hosokawa M., Shoji M., Kitamura K. (2007). Tudor-related proteins TDRD1/MTR-1, TDRD6 and TDRD7/TRAP: domain composition, intracellular localization, and function in male germ cells in mice. *Developmental Biology*.

[10] Tanaka T., Hosokawa M., Vagin V. V. (2011). Tudor domain containing 7 (Tdrd7) is essential for dynamic ribonucleoprotein (RNP) remodeling of chromatoid bodies during spermatogenesis. *Proceedings of the National Academy of Sciences*.

[11] Iliev R., Fedorko M., Machackova T. (2016). Expression levels of PIWI-interacting RNA, piR-823, are deregulated in tumor tissue, blood serum and urine of patients with renal cell carcinoma. *Anticancer Research*.

[12] Zhao C., Tolkach Y., Schmidt D. (2019). Mitochondrial PIWI-interacting RNAs are novel biomarkers for clear cell renal cell carcinoma. *World Journal of Urology*.

[13] Fang L., Zhang M., Chen L. (2016). Downregulation of nucleolar and spindle-associated protein 1 expression suppresses cell migration, proliferation and invasion in renal cell carcinoma. *Oncology Reports*.

[14] Li Y., Wu X., Gao H. (2015). Piwi-interacting RNAs (piRNAs) are dysregulated in renal cell carcinoma and associated with tumor metastasis and cancer-specific survival. *Molecular Medicine*.

[15] Busch J., Ralla B., Jung M. (2015). Piwi-interacting RNAs as novel prognostic markers in clear cell renal cell carcinomas. *Journal of Experimental & Clinical Cancer Research*.

[16] Chen C., Nott T. J., Jin J., Pawson T. (2011). Deciphering arginine methylation: Tudor tells the tale. *Nature Reviews. Molecular Cell Biology*.

[17] (2012). _Current Biology_. *Current Biology*.

[18] Chen C., Jin J., James D. A. (2009). Mouse Piwi interactome identifies binding mechanism of Tdrkh Tudor domain to arginine methylated Miwi. *Proceedings of the National Academy of Sciences*.

[19] Wang J., Saxe J. P., Tanaka T., Chuma S., Lin H. (2009). Mili interacts with tudor domain-containing protein 1 in regulating spermatogenesis. *Current Biology*.

[20] Pandey R. R., Tokuzawa Y., Yang Z. (2013). Tudor domain containing 12 (TDRD12) is essential for secondary PIWI interacting RNA biogenesis in mice. *Proceedings of the National Academy of Sciences of the United States of America*.

[21] Pan J., Goodheart M., Chuma S., Nakatsuji N., Page D. C., Wang P. J. (2005). RNF17, a component of the mammalian germ cell nuage, is essential for spermiogenesis. *Development*.

[22] Patil V. S., Anand A., Chakrabarti A., Kai T. (2014). The Tudor domain protein Tapas, a homolog of the vertebrate Tdrd7, functions in the piRNA pathway to regulate retrotransposons in germline of Drosophila melanogaster. *BMC Biology*.

[23] Mo H. Y., Choi E. J., Yoo N. J., Lee S. H. (2020). Mutational alterations of TDRD 1, 4 and 9 genes in colorectal cancers. *Pathology Oncology Research*.

[24] Li D., Liu S., Xu J. (2021). Ferroptosis-related gene CHAC1 is a valid indicator for the poor prognosis of kidney renal clear cell carcinoma. *Journal of Cellular and Molecular Medicine*.

[25] Xu W. H., Xu Y., Wang J. (2019). Prognostic value and immune infiltration of novel signatures in clear cell renal cell carcinoma microenvironment. *Aging (Albany NY)*.

[26] Mou Y., Wu J., Zhang Y., Abdihamid O., Duan C., Li B. (2021). Low expression of ferritinophagy-related NCOA4 gene in relation to unfavorable outcome and defective immune cells infiltration in clear cell renal carcinoma. *BMC Cancer*.

[27] Xing S., Yu W., Zhang X. (2018). Isoviolanthin extracted from Dendrobium officinale reverses TGF-*β*1-Mediated Epithelial–Mesenchymal transition in hepatocellular carcinoma cells via deactivating the TGF-*β*/Smad and PI3K/Akt/mTOR Signaling pathways. *International Journal of Molecular Sciences*.

