# Supplementary material for: PIWI-Interacting RNA Pathway Genes: Potential Biomarkers for Clear Cell Renal Cell Carcinoma
Source: Dis Markers. 2022 Mar 1;2022:3480377. doi: 10.1155/2022/3480377 (PMC8904100; doi:10.1155/2022/3480377)
Supplement: Supplementary Materials — Table S1: univariate and multivariate Cox regression analysis of clinical factors and the piRNA pathway gene risk score for overall survival. [file 3480377.f1.docx]

**Supplementary Table**

**Table S1. Univariate and multivariate Cox regression analysis of clinical factors and the piRNA pathway gene risk score for overall survival.**

| Variable | Overall survival | | | | |
| --- | --- | --- | --- | --- | --- |
|  | Univariate | |  | Multivariate | |
|  | HR (95% CI) | P |  | HR (95% CI) | P |
| Age |  |  |  |  |  |
| ≥60 *vs*. <60 | 1.175(0.629-2.196) | 0.612 |  | 1.683(0.747-3.791) | 0.209 |
| Gender |  |  |  |  |  |
| Male *vs*. female | 1.161(0.630-2.141) | 0.633 |  | 1.192(0.592-2.400) | 0.623 |
| Stage |  |  |  |  |  |
| III–IV *vs*. I–II | 2.699(1.454-5.010) | **0.002** |  | 0.693(0.133-3.607) | 0.663 |
| Grade |  |  |  |  |  |
| 3+4 *vs*. 1+2 | 1.809(0.940-3.482) | 0.076 |  | 1.086(0.514-2.295) | 0.829 |
| T |  |  |  |  |  |
| T3–T4 *vs*. T1–T2 | 2.279(1.240-4.187) | **0.008** |  | 1.930(0.472-7.897)) | 0.360 |
| N |  |  |  |  |  |
| N1 *vs* N0 | 2.324(0.716-7.547) | 0.160 |  | 3.283(0.676-15.934) | 0.140 |
| Metastasis |  |  |  |  |  |
| M1 *vs*. M0 | 6.698(3.119-14.384) | **< 0.001** |  | 6.924(2.346-20.437) | **< 0.001** |
| Risk score |  |  |  |  |  |
| High *vs*. Low | 1.430(1.197-1.708) | **< 0.001** |  | 1.252(1.006-1.557) | **0.044** |
